# Cognitive evaluation in Parkinson's disease: applying the Movement Disorder Society recommendations in a population with a low level of formal education

**DOI:** 10.1055/s-0042-1759761

**Published:** 2023-03-22

**Authors:** Natalia Pessoa Rocha, Eduarda Xavier Carreira, Ana Carolina de Almeida Prado, Fabíola Tavares, Mayra Tavares, Francisco Cardoso, Antônio Jaeger, Leonardo Cruz de Souza, Antônio Lucio Teixeira

**Affiliations:** 1 Universidade Federal de Minas Gerais, Faculdade de Medicina, Laboratório Interdisciplinar de Investigação Médica, Belo Horizonte MG, Brazil.; 2 The University of Texas Health Science Center at Houston, McGovern Medical School, Houston, Department of Psychiatry and Behavioral Sciences, Neuropsychiatry Program, Texas, United States.; 3 Universidade Federal de Minas Gerais, Faculdade de Filosofia e Ciências Humanas, Departamento de Psicologia, Belo Horizonte MG, Brazil.; 4 Universidade Federal de Minas Gerais, Serviço de Neurologia, Unidade de Distúrbios do Movimento, Belo Horizonte MG, Brazil.; 5 Universidade Federal de Minas Gerais, Faculdade de Medicina, Departamento de Medicina Interna, Belo Horizonte MG, Brazil.

**Keywords:** Parkinson Disease, Cognitive Dysfunction, Dementia, Neuropsychological Tests, Educational Status, Doença de Parkinson, Disfunção Cognitiva, Demência, Testes Neuropsicológicos, Escolaridade

## Abstract

**Background** The diagnosis of cognitive disorders in Parkinson disease (PD) can be very challenging. Aiming at establishing uniform and reliable diagnostic procedures, the International Parkinson's Disease and Movement Disorder Society (MDS) commissioned task forces to delineate diagnostic criteria for mild cognitive impairment (MCI) and dementia in PD.

**Objectives** To investigate the applicability of the MDS recommendations for cognitive evaluation in a Brazilian sample of patients with PD with low levels of formal education.

**Methods** A total of 41 patients with PD were subjected to a comprehensive neuropsychological evaluation based on tests proposed by the MDS, which included the Mini-Mental State Examination, the Mattis Dementia Rating Scale (MDRS), the Trail Making Test (TMT) parts A and B, in addition to language and memory skills assessment. Neuropsychiatric and daily functioning features were also evaluated. Spearman correlation analyses were used to evaluate the association between the scores obtained in the cognitive scales and demographic/clinical variables.

**Results** Although none of the participants had a formal diagnosis of dementia, 50% presented some degree of cognitive impairment when considering the results of the MDRS. Of note, a noticeable number of patients was not able to complete the full neuropsychological assessment. The TMT part B was the most difficult task, being completed by only 22 participants (54%). As expected, the greater the educational level, the better the performance on the cognitive tests. Better motor function was also associated with better scores in cognition.

**Conclusions** Adopting strict inclusion/exclusion criteria and a comprehensive clinical evaluation, we found remarkable limitations for the MDS recommendations when individuals with low educational levels are considered. A revision of the current guidelines is necessary considering differences among populations, especially related to formal education.

## Resumo

**Antecedentes** O diagnóstico de distúrbios cognitivos na doença de Parkinson (DP) pode ser muito desafiador. Com o objetivo de estabelecer procedimentos diagnósticos uniformes e confiáveis, a Sociedade Internacional da Doença de Parkinson e Distúrbios do Movimento (MDS, na sigla em inglês) encomendou forças-tarefa para delinear critérios diagnósticos para comprometimento cognitivo leve (CCL) e demência na DP.

**Objetivos** Investigar a aplicabilidade das recomendações da MDS para avaliação cognitiva em uma amostra brasileira de pacientes com DP de baixa escolaridade.

**Métodos** Um total de 41 pacientes com DP foram submetidos a uma avaliação neuropsicológica abrangente com base nos testes propostos pela MDS, que incluiu o Miniexame do Estado Mental, a Escala de Avaliação de Demência de Mattis (MDRS, na sigla em inglês), o teste de trilhas (TMT, na sigla em inglês) partes A e B, além da avaliação das habilidades de linguagem e memória. Características neuropsiquiátricas e de funcionamento diário também foram avaliadas. Análises de correlação de Spearman foram utilizadas para avaliar a associação entre os escores obtidos nas escalas cognitivas e variáveis demográficas/clínicas.

**Resultados** Apesar de nenhum dos participantes ter diagnóstico formal de demência, 50% apresentaram algum grau de comprometimento cognitivo ao levar em consideração os resultados da MDRS. Vale ressaltar que um número notável de pacientes não conseguiu completar a avaliação neuropsicológica completa. A parte B do TMT foi a tarefa mais difícil, sendo realizada por apenas 22 participantes (54%). Como esperado, quanto maior o nível educacional, melhor o desempenho nos testes cognitivos. Melhor função motora também foi associada a melhores escores em cognição.

**Conclusões** Adotando critérios rígidos de inclusão/exclusão e uma avaliação clínica abrangente, encontramos limitações marcantes para as recomendações da MDS quando considerados indivíduos com baixa escolaridade. É necessária uma revisão das diretrizes atuais considerando as diferenças entre as populações, principalmente relacionadas ao nível educacional.

## INTRODUCTION

Cognitive impairment in Parkinson disease (PD) is a heterogeneous condition regarding pattern, severity, and progression. Different neuropsychological domains can be affected, but most patients with PD exhibit a certain degree of executive dysfunction. The cognitive impairment in PD can vary from mild cognitive impairment (MCI) – when symptoms are not sufficient to affect activities of daily living – to severe dementia. 1 Mild cognitive impairment in PD (PD-MCI) may be a harbinger of PD dementia (PD-D), which can occur in up to 80% of patients with PD over the long term (8–20 years). 2

The diagnosis of either PD-MCI or PD-D and the identification of the pattern of cognitive impairment are relevant due to their prognostic value. 1 Cognitive impairment in PD can be categorized into single-domain, multiple-domain, amnestic, and nonamnestic subtypes, based on the results of neuropsychological testing. 2 Moreover, a neuropsychological evaluation is usually required to select patients with PD eligible for specific treatments, such as deep brain stimulation. 3 In this scenario, establishing uniform and reliable diagnostic procedures for both PD-MCI and PD-D is particularly important, and will contribute to the identification of: i) cognitive impairment and its characteristics since the early stages of the disease; ii) the best predictors of conversion from PD-MCI to PD-D; and iii) potential outcome measures for clinical trials. Specific criteria for diagnosing MCI and dementia in PD can also improve patient care and research efforts. 2 4

With these perspectives in mind, the International Parkinson's Disease and Movement Disorder Society (MDS) commissioned task forces to delineate diagnostic criteria for MCI 2 5 and dementia 4 6 in PD. These working groups proposed a set of neuropsychological tests for assessing PD-related cognitive deficits in clinical settings. After the MDS proposals, great advances have occurred in the field of cognitive assessment in PD. Nevertheless, studies that addressed the validity of the proposed cognitive assessment in PD yielded mixed results, and most concluded that the accuracy of the instruments was not entirely satisfactory. 7 8 9 10

A critical point for cognitive batteries with diagnostic purposes is the definition of cutoffs according to the educational level of the patients. Because education is a major factor underlying cognitive performance, 11 the selection of cognitive tests must always consider formal schooling. Unfortunately, several cognitive tests are not suitable for populations with low levels of formal education, which frequently exhibit floor effects on these tests. While cognitive tools are well suited for diagnosing cognitive deficits in educated individuals, their clinical value for illiterate patients or those with few years of formal education remains debatable. So far, no study has investigated the diagnostic value of the MDS recommendations for populations with low levels of formal education. This is of utmost importance as there is an increase of patients with neurodegenerative diseases in low-income countries, and due to the immigration of populations with low levels of formal education to high-income nations. Herein, we aimed to investigate the applicability of the MDS recommendations for cognitive evaluation in a Brazilian sample of patients with PD with a low level of formal education.

## METHODS

### Subjects

This is a cross-sectional study that consecutively included 41 patients with PD diagnosed according to the United Kingdom PD Brain Bank criteria. 12 Patients were recruited from the Movement Disorders Unity of the Neurology Service of the Universidade Federal de Minas Gerais, Belo Horizonte, state of Minas Gerais, Brazil, from May 2015 until July 2016. In order to increase diagnosis accuracy, we only recruited patients with > 5 years of disease (formal clinical diagnosis). Participants were excluded if they had undergone previous neurosurgery or if they had any other neurological or severe psychiatric disorders (e.g., psychosis) or significant sensory impairment. Participants were also excluded if they had a formal diagnosis of depression, or anxiety, or if they were using anticholinergic or sedative drugs at the time of recruitment. All subjects, or their relatives when appropriate, provided written informed consent before enrollment in the study.

The present study was approved by the Committee for the Protection of Human Subjects of the Universidade Federal de Minas Gerais (Ref. #: CAAE 0417.0.203.000–11). All subjects, or their relatives when appropriate, provided written informed consent before enrollment in the study.

The data that support the findings of the present study are available from the corresponding author upon reasonable request.

### Clinical evaluation

All clinical assessments were performed in a single session, during the “on” state. Clinical assessment included the Unified Parkinson's Disease Rating Scale (UPDRS), 13 which evaluates different PD-related signs and symptoms, and a comprehensive neuropsychological evaluation, which included cognitive tests proposed by the MDS task forces for MCI 2 5 and dementia 4 diagnoses. According to the MDS recommendations, cognitive examination in PD should include a global cognition assessment and at least one test per cognitive domain, that is, attention/working memory, executive function, language, memory, and visuospatial function. To specify the pattern and severity of the cognitive impairment once it is established, it is necessary the application of at least one further test for each cognitive domain. 2 4 5

Global cognition was evaluated by the Mini-Mental State Examination (MMSE). 14 The Mattis Dementia Rating Scale (MDRS) 15 was also applied since it provides a more comprehensive mental status examination, contributing to characterizing the severity of the cognitive impairment (MCI or dementia) and its relation to the dysexecutive syndrome, a key feature of cognitive impairment in PD. 4

The Trail Making Test (TMT) parts A and B were used for attention/working memory and executive function evaluation, respectively. 16 Language was assessed by the category fluency test (animal naming). 17 For memory evaluation (such as free and cued selective reminding test or figural memory learning and delayed recall), 5 we used the 10 black and white pictures of the Brief Cognitive Screening Battery. 18 Although this test is not in the MDS list, it corresponds to the recommended tests, and evaluates incidental memory, short-term memory, learning and delayed memory of 10 figures (after 5 minutes), and recognition of 10 figures. Moreover, it has been validated in the Brazilian population and proved to be suitable for the assessment of memory in populations with a high frequency of illiterates or low educational level individuals. 18

Behavioral disorders were evaluated through the Neuropsychiatric Inventory (NPI). 19 Using the information obtained from a caregiver familiar with the patient's behavior, the NPI uses a screening strategy to score the following behavioral domains: delusions, hallucinations, depression/dysphoria, anxiety, agitation/aggression, elation/euphoria, disinhibition, irritability/lability, apathy, aberrant motor activity, nighttime behaviors, and appetite/eating. The frequency and severity of the symptoms are determined for those behaviors with positive responses to the screening questions. 19 The Brazilian version of the apathy scale directed to the caregiver was also applied to evaluate apathy symptoms. The apathy scale scores range from 0 to 42, with higher scores indicating greater apathy, and a score ≥ 14 is indicative of clinically meaningful apathy in PD. 20 21 In addition, all participants were evaluated using the Beck Depression Inventory (BDI), a self-rating instrument for depressive symptoms comprising 21 items, each one ranging from 0 to 3 according to the severity of symptoms. 22 The BDI has been validated as a tool for depression screening and diagnosis in PD, including Brazilian patients. A score ≥ 18 was used to recognize depression in our sample. 23

The diagnosis of dementia requires both the presence of significant cognitive changes and the subsequent impact on activities of daily living (ADL). The Brazilian version of the Pfeffer Functional Assessment Questionnaire (FAQ) was used to assess functional status. 24 25 Based on the FAQ scores, the functionality of the participants was categorized into 3 groups: functionally intact (total FAQ scores = 0 to 5), mild (scores = 6 to 10), and moderate to severe (scores = 11 to 30).

### Statistical analyses

The results of the scores obtained in the clinical scales are presented as mean and standard deviation (SD) and median. For categorical variables, the results are presented either as absolute numbers or percentages. Spearman correlation analyses were used to evaluate the association between the scores obtained in the cognitive scales and demographic/clinical variables (age, disease length, educational level, UPDRS scores, and scores in the neuropsychiatric/functionality scales).

## RESULTS

### General characteristics and cognitive evaluation

Forty-one patients with PD participated in the study. The general features of the participants and the results of the cognitive evaluation are shown in Table 1 . Although our sample was composed of patients with long-term disease course (mean 11.21 years), motor symptoms were well controlled, since they showed mild to moderate impairment at the UPDRS (mean 41.61).

**Table 1:** General and clinical features of patients with Parkinson disease

General features:	
Gender (female/male)	15/26
Age (years old) (mean ± SD [median])	64.44 ± 9.72 (65)
Length of illness in years (mean ± SD [median])	11.21 ± 6.07 (10)
Educational level in years (mean ± SD [median])	7.15 ± 4.32 (6.5)
UPDRS total score (mean ± SD [median])	41.61 ± 22.15 (36)
UPDRS I (mean ± SD [median])	4.89 ± 4.52 (4)
UPDRS II (mean ± SD [median])	12.89 ± 9.01 (11)
UPDRS III (mean ± SD [median])	23.83 ± 13.61 (20)
**Cognitive evaluation:**	
MMSE (mean ± SD [median])	24.21 ± 4.01 (25)
MDRS total score (mean ± SD [median])	123.09 ± 15.79 (126.5)
MDRS - Attention	34.10 ± 3.51 (35)
MDRS - Initiation	30.03 ± 6.75 (33)
MDRS - Construction	4.54 ± 1.95 (6)
MDRS - Conceptualization	31.29 ± 6.41 (32)
MDRS - Memory	21.18 ± 4.45 (23)
TMT-A, time in seconds (mean ± SD [median])	97.44 ± 50.48 (89)
TMT-A, errors (mean ± SD [median])	0.18 ± 0.72 (0)
TMT-B, time in seconds (mean ± SD [median])	250.68 ± 112.59 (220)
TMT-B, errors (mean ± SD [median])	0.77 ± 1.41 (0)
Category fluency test, animal naming (mean ± SD [median])	13.50 ± 4.19 (13.5)
10-picture test, incidental memory (mean ± SD [median])	4.43 ± 1.70 (5)
10 pictures test, immediate memory (mean ± SD [median])	7.00 ± 1.96 (7)
10-picture test, learning (mean ± SD [median])	7.91 ± 1.60 (8)
10-picture test, recognition (mean ± SD [median])	9.60 ± 0.70 (10)
10-picture test, delayed recall (mean ± SD [median])	6.74 ± 2.44 (7)

Abbreviations: MDRS, Mattis Dementia Rating Scale; MMSE, Mini-Mental State Examination; PD, Parkinson disease; SD, standard deviation; TMT, Trail Making Test; UPDRS, Unified Parkinson's Disease Rating Scale.

None of the participants had a formal diagnosis of dementia. Overall, participants had preserved global cognition as assessed by the MMSE, with a mean score of 24. This value was significantly above the cutoff score established for individuals with a low level of formal education, as the current participants (MMSE = 18). 26 Only 6 participants (15%) scored below the MMSE cutoff established for their educational level. 26 However, when taking into account the results from the MDRS, a higher percentage of patients (50%) presented some degree of cognitive impairment. The mean MDRS score for the whole sample (123.09) coincided with the value established as a cutoff for this scale in PD patients (MDRS ≤ 123). 27 The most compromised ability was Construction (44% of patients scored inferior to normative data), followed by Initiation/Perseveration (33% scored inferior). Regarding memory tests, only 2 (5%) participants presented memory deficits, that is, scored < 5 in the delayed recall task of the 10-picture test. 28 When considering the normative data for memory deficits in the MDRS, 26% of participants scored below the average.

Surprisingly, a noticeable number of patients was not even able to complete the full neuropsychological assessment. The TMT part B was the most difficult task, being completed by only 22 participants (54%). In addition, the mean time spent to complete this task was of 250.68 seconds, significantly longer than the expected time for the general population, 29 and for a Brazilian control sample within the same age and educational level. 30 A higher number of participants ( *n* = 34; 83%) was capable of completing the TMT-A, spending on average 97.44 seconds to complete the task, although the performance was again worse than other populations in the same age range. 31

### Neuropsychiatric and daily functioning features

The results obtained in the neuropsychiatric and daily functioning assessments are shown in Table 2 . Patients with PD exhibited a range of neuropsychiatric symptoms, but overall, these symptoms were not severe. According to the NPI, nighttime behaviors were the most common neuropsychiatric symptoms in our sample (57%), followed by depression/dysphoria (52%), apathy (38%), and anxiety (38%). Of note, 31% of the participants were considered depressed according to the BDI (≥ 18), and 44% presented clinical meaningful apathy, based on the apathy scale (≥ 14). Finally, most patients presented functional independence, according to Pfeffer FAQ. Specifically, 71% of patients were considered functionality intact, 17% presented mild impairment, and 13% moderate to severe impairment. It is worth mentioning that Pfeffer FAQ assesses functionality from cognitive rather than motor skills.

**Table 2:** Neuropsychiatric and daily functioning features

	Mean ± SD (median)	% of patients who scored ≥ 1
NPI	26.67 ± 24.26 (24)	95
Delusions	2.29 ± 4.96 (0)	24
Hallucinations	2.38 ± 4.85 (0)	33
Agitation/Aggression	1.52 ± 3.70 (0)	19
Depression/Dysphoria	2.81 ± 3.91 (1)	52
Anxiety	3.19 ± 5.11 (0)	38
Elation/Euphoria	0.33 ± 1.53 (0)	5
Apathy/Indifference	2.24 ± 3.43 (0)	38
Disinhibition	1.38 ± 3.31 (0)	19
Irritability/Lability	0.67 ± 1.74 (0)	14
Motor Disturbance	1.29 ± 3.00 (0)	24
Nighttime Behaviors	5.71 ± 6.13 (6)	57
Appetite/Eating	2.86 ± 5.20 (0)	38
BDI, mean ± SD (median)	14.12 ± 10.83 (14)	
Apathy scale, mean ± SD (median)	13.28 ± 8.91 (13)	
Pfeffer's FAQ, mean ± SD (median)	5.00 ± 7.55 (2)	
Functionally intact (0–5)	71%	
Mild impairment (6–10)	17%	
Moderate to severe impairment (11–30)	13%	

Abbreviations: BDI, Beck Depression Inventory; FAQ, Functional Activities Questionnaire; NPI, Neuropsychiatric Inventory; PD, Parkinson disease; SD, standard deviation.

### Association between cognitive measurements and clinical data

As expected, the greater the educational level, the better the performance on the MMSE, MDRS (total scores), TMT-A, TMT-B, and the category fluency test (animal naming). In contrast, the memory scores obtained in the '10 pictures test' were not correlated with years of education ( Table 3 ). Better motor function, as measured by the UPDRS, was often associated with better scores in cognition. Specifically, motor function was associated with global cognition (MMSE and MDRS), attention/working memory (TMT-A), and incidental memory (10-picture test). Motor function was also associated with executive functions (TMT-B), although this correlation was only marginally significant (rho = 0.426; *p* = 0.069 ( Table 3 ).

**Table 3:** Correlations between cognitive measures and clinical data

		Age	Disease length	Educational level	UPDRS total score	UPDRS I	UPDRS II	UPDRS III
MMSE	rho	−.281	−0.160	**0.533**	**−0.425**	**−0.421**	**−0.410**	**−0.419**
	***p-value***	0.083	0.344	**0.000**	**0.012**	**0.013**	**0.016**	**0.014**
MDRS total score	rho	−0.112	−0.290	**0.350**	**−0.660**	−0.306	**−0.559**	**−0.659**
	***p-value***	0.530	0.108	**0.043**	**0.000**	0.101	**0.001**	**0.000**
MDRS - Attention	rho	−0.234	−0.129	0.225	−0.245	−0.241	−0.212	−0.240
	***p-value***	0.151	0.445	0.169	0.162	0.170	0.229	0.172
MDRS - Initiation	rho	**−0.386**	−0.163	0.219	**−0.665**	−0.185	**−0.649**	**−0.661**
	***p-value***	**0.015**	0.336	0.180	**0.000**	0.296	**0.000**	**0.000**
MDRS - Construction	rho	−0.152	−0.254	**0.337**	**−0.388**	−0.326	**−0.341**	**−0.386**
	***p-value***	0.355	0.129	**0.036**	**0.023**	0.060	**0.048**	**0.024**
MDRS - Conceptualization	rho	0.129	**−0.427**	**0.382**	**−0.508**	**−0.521**	**−0.328**	**−0.471**
	***p-value***	0.460	**0.013**	**0.023**	**0.004**	**0.003**	**0.072**	**0.007**
MDRS - Memory	rho	−0.275	−0.118	0.149	**−0.398**	**−0.485**	**−0.362**	**−0.368**
	***p-value***	0.095	0.494	0.373	**0.022**	**0.004**	**0.039**	**0.035**
TMT-A (time)	rho	0.287	0.134	**−0.406**	**0.372**	0.013	**0.535**	0.353
	***p-value***	0.100	0.463	**0.017**	**0.043**	0.947	**0.002**	0.056
TMT-B (time)	rho	0.116	0.329	**−0.421**	0.426	0.359	0.388	0.246
	***p-value***	0.609	0.146	**0.050**	0.069	0.131	0.101	0.309
Category fluency test, animal naming	rho	−0.147	−0.014	**0.493**	−0.207	−0.029	−0.276	−0.185
	***p-value***	0.378	0.936	**0.002**	0.247	0.874	0.120	0.303
Incidental memory	rho	−0.037	−0.232	0.104	**−0.415**	**−0.519**	**−0.386**	0.213
	***p-value***	0.834	0.194	0.551	**0.023**	**0.003**	**0.035**	0.257
Immediate memory	rho	**−0.465**	0.076	0.285	−0.270	−0.082	−0.201	−0.288
	***p-value***	**0.005**	0.673	0.097	0.149	0.666	0.286	0.122
Learning	rho	**−0.545**	0.136	0.233	−0.223	−0.078	−0.219	−0.280
	***p-value***	**0.001**	0.452	0.178	0.235	0.683	0.246	0.135
Recognition	rho	−0.161	−0.059	−0.098	−0.226	−0.159	−0.131	−0.233
	***p-value***	0.356	0.746	0.575	0.229	0.400	0.489	0.216
Delayed recall	rho	**−0.418**	−0.155	0.123	−0.179	0.124	−0.153	−0.327
	***p-value***	**0.012**	0.388	0.483	0.344	0.515	0.421	0.078

Abbreviations: MDRS, Mattis Dementia Rating Scale; MMSE, Mini-Mental State Examination; TMT, Trail Making Test; UPDRS, Unified Parkinson's Disease Rating Scale.

Note: Significant correlations are highlighted in bold.

Regarding neuropsychiatric symptoms, higher scores in the NPI were associated with worse performance in the MDRS memory tasks, and with worse performance in the recognition task of the '10 pictures test'. Also, higher BDI scores correlated with a longer time to complete the TMT-B and lower scores in the incidental memory of the 10 pictures test. Apathy symptoms correlated with MMSE and the MDRS-construction scores ( Table 4 ). Finally, worse daily functioning was associated with worse performance in most cognitive measurements: MMSE, MDRS and subitems, TMT-A, TMT-B, category fluency, immediate memory, and learning from the 10-pictures test ( Table 4 ).

**Table 4:** Correlations between cognitive measures and neuropsychiatry/functionality data

		NPI	BDI	Apathy Scale	Pfeffer FAQ
MMSE	rho	−0.365	−0.169	**−0.421**	**−0.563**
	***p-value***	0.104	0.348	**0.036**	**0.003**
MDRS total score	rho	−0.359	−0.191	−0.274	**−0.697**
	***p-value***	0.157	0.312	0.229	**0.000**
MDRS - Attention	rho	0.056	0.024	0.021	−0.356
	***p-value***	0.809	0.893	0.920	0.081
MDRS - Initiation	rho	−0.362	−0.036	−0.185	**−0.710**
	***p-value***	0.107	0.842	0.377	**0.000**
MDRS - Construction	rho	−0.279	−0.076	**−0.410**	**−0.387**
	***p-value***	0.221	0.676	**0.042**	**0.056**
MDRS - Conceptualization	rho	−0.450	−0.326	−0.418	**−0.428**
	***p-value***	0.061	0.079	0.053	**0.047**
MDRS - Memory	rho	**−0.534**	−0.299	−0.296	**−0.497**
	***p-value***	**0.015**	0.091	0.160	**0.004**
TMT-A (time)	rho	0.044	0.211	0.202	**0.738**
	***p-value***	0.858	0.246	0.366	**0.000**
TMT-B (time)	rho	0.119	**0.517**	0.150	**0.602**
	***p-value***	0.712	**0.016**	0.609	**0.023**
Category fluency test, animal naming	rho	−0.158	−0.188	−0.291	**−0.498**
	***p-value***	0.507	0.295	0.167	**0.013**
Incidental memory	rho	−0.432	**−0.497**	−0.278	−0.364
	***p-value***	0.065	**0.004**	0.211	0.096
Immediate memory	rho	0.117	−0.253	0.060	**−0.588**
	***p-value***	0.632	0.163	0.789	**0.004**
Learning	rho	0.176	−0.107	0.059	**−0.473**
	***p-value***	0.471	0.559	0.795	**0.026**
Recognition	rho	**−0.573**	0.060	−0.227	0.003
	***p-value***	**0.010**	0.743	0.310	0.990
Delayed recall	rho	−0.184	−0.131	−0.073	−0.370
	***p-value***	0.451	0.474	0.747	0.090

Abbreviations: BDI, Beck Depression Inventory; FAQ, Functional Activities Questionnaire; MDRS, Mattis Dementia Rating Scale; MMSE, Mini-Mental State Examination; NPI, Neuropsychiatric Inventory; TMT, Trail Making Test.

Note: Significant correlations are highlighted in bold.

## DISCUSSION

Cognitive impairment is very common in patients with PD, and dementia in PD is associated with a significant increase in mortality. 32 Yet, overall cognitive deficiencies in PD remain underdiagnosed and undertreated. The multifaceted cognitive phenotype in PD makes the diagnosis of cognitive disorders very challenging in this population. 33 Given that, the MDS defined the diagnostic criteria for MCI 2 5 and dementia 4 6 in PD, including recommendations of specific neuropsychological tests for assessing PD-related cognitive deficits in clinical settings. However, the guidelines have not addressed limitations inherent to populations with low levels of formal education.

In the present study, we applied the MDS recommendations for cognitive evaluation in a Brazilian sample of patients with PD with a low level of formal education. However, we found that the battery of tests was not appropriate for our population, as several participants (46%) were not even able to complete it. Accordingly, a revision of the current guidelines is necessary considering differences among populations, especially related to educational level.

The low level of education observed in our sample (median = 6.5 years) mirrors the Brazilian population. In 2016, people ≥ 60 years old in Brazil presented a mean educational level of 6.0 years and 20.4% of these individuals were illiterate. 34 In this context, as the performance in most cognitive tests depends on the educational level, the cutoffs for cognitive impairment proposed by international guidelines are not appropriate when applied to Brazilian and populations of other developing countries. 30

Interestingly, a lower educational level is associated not only with poorer performance in cognitive tests but also with the potential development of cognitive impairment in PD. For instance, Kierzynka et al. showed that patients with PD had worse cognitive performance than controls in a comprehensive battery including the TMT-A, the TMT-B, the verbal fluency test, the Stroop, and the MMSE. Differences between patients and controls were observed in subjects with low and intermediate educational levels. However, no significant differences were observed between highly educated PD patients and matched controls, suggesting that more advanced education may foster a protective effect against early cognitive decline in PD. 35 These findings corroborate previous studies that demonstrated the protective effects of education and cognitive reserve on cognitive aging and reduced risk of dementia, 11 36 including reports involving patients with PD. 37 38

The mean score obtained in the MMSE was considerably higher than the cutoff score established for the educational level of our sample. Considering the MMSE score for cognitive impairment screening, only 6 participants (15%) scored below the cutoff established for their educational level. 26 Although the MMSE is an important tool for global cognitive assessment and screening for cognitive impairment/dementia, its utility in PD has been questioned, especially given its overemphasis on language and orientation, which are relatively preserved in PD. Moreover, the MMSE was found to miss ∼ 55% of cases of dementia in PD. 39 These findings highlight the clinical value of a more comprehensive neuropsychological assessment for the diagnosis of cognitive impairment and dementia in PD. Accordingly, when considering the results from the MDRS, half of our patients exhibited some degree of cognitive impairment. It is worth mentioning that the cutoff score in the MDRS for screening dementia in PD 27 has also been debatable, 40 highlighting the relevance of using multiple tests or tools for a diagnosis of cognitive impairment. In the current study, we conducted a neuropsychological assessment following the MDS recommendations, adopting the TMT-A and TMT-B as part of our evaluation. These tests have been ranked as first-line tools to assess attention/working memory and executive function in PD, respectively. 5 The current findings, however, suggest that the TMT is not appropriate for individuals with low levels of formal education, since only 22 out of our 41 patients (54%) were able to complete the TMT-B, and there was no association between performance on the TMT with attention and executive (as measured by the MDRS). Failure to complete the test may also be related to executive impairment, mainly alternating attention, and visuomotor speed. Furthermore, although the TMT is considered a test of visual search, attention, mental flexibility, and motor function, its scores were affected by motor speed, age, and educational level. In addition, significant differences were found in the TMT-B normative data among different countries and cultures, a fact that might lead to diagnostic errors. 29

Adopting strict inclusion/exclusion criteria and a comprehensive clinical evaluation, we found remarkable limitations for the MDS recommendations when individuals with low levels of formal education are considered. The current cross-sectional design, the small sample size, and the lack of a control group, however, prevented us from performing a more sophisticated statistical analysis. Also, because all patients were on medication, the question remains of whether this treatment affected the performance of the individuals on the tests. The fact that the patients had to complete all the assessments in one single session may also influence their performance, as patients with PD often experience fatigue. Finally, although the patients did not have a formal diagnosis of psychiatric diseases at the time of recruitment, they presented with neuropsychiatric symptoms, and a good percentage of participants scored above the cutoff for depression (31%) and clinical meaningful apathy (44%). The presence of neuropsychiatric symptoms can interfere with the performance of patients on the cognitive tests. These questions should be considered in future studies.

In conclusion, our results show that although they did not have a formal diagnosis of MCI or dementia, half of the individuals with PD included in our study exhibited some degree of cognitive impairment. In addition, a good number of participants was not able to complete the full neuropsychological assessment as proposed by the MDS. While our results may be influenced by other comorbidities such as depression, formal educational level impacts directly the ability to complete the tasks proposed by the MDS. Therefore, the current guidelines should be revised to incorporate differences among populations, especially related to formal education.
